# A global dataset of average specific yield for soils

**DOI:** 10.1038/s41597-025-04742-1

**Published:** 2025-03-12

**Authors:** Meizhao Lv, Meixia Lv, Yuanyuan Zha, Lei Wang, Zong-Liang Yang

**Affiliations:** 1 CAS Key Laboratory of Regional Climate and Environment for Temperate East Asia, Institute of Atmospheric Physics, Chinese Academy of Sciences, Beijing, China; 2 State Key Laboratory of Water Resources Engineering and Management, Wuhan University, Wuhan, China; 3 State Key Laboratory of Tibetan Plateau Earth System, Environment and Resources, Institute of Tibetan Plateau Research, Chinese Academy of Sciences, Beijing, China; 4 The University of Chinese Academy of Sciences, Beijing, China; 5 Department of Earth and Planetary Sciences, Jackson School of Geosciences, The University of Texas at Austin, Austin, TX 78705 USA

**Keywords:** Hydrology, Climate and Earth system modelling

## Abstract

Specific yield (*S*_y_) stands as a critical parameter and a significant source of error in groundwater simulations. However, there is still a lack of reliable global *S*_y_ datasets. Based on the trilinear graph of *S*_y_ and soil textures, we develop a comprehensive global dataset of gridded average specific yield (GASY) aimed for various soil textures, which are obtained from the Global Soil Dataset for Earth System Models, the SoilGrids product, and the Harmonized World Soil Database. Validations with existing *S*_y_ values estimated by laboratory and field methods across different *S*_y_ concepts, at the aquifer-scale to global-scale, compellingly revealed that the GASY effectively represents reliable average *S*_y_ for each soil texture. The depth limitation (~2 m) of GASY is attributed to the depth limitations of soil texture data, and readers can expand the GASY into deeper soils by reasonably assuming a vertical variation of soil texture with depth. The GASY holds great benefits for future modeling of groundwater dynamics and understanding the groundwater resources distribution and mitigation of climate change impacts.

## Background & Summary

Specific yield (*S*_y_ [-]) is pivotal in groundwater researches, delineating the capacity for groundwater gain or loss with water table fluctuations. It serves as a key factor in converting groundwater storage changes (GWSC) to groundwater level changes (GWLC) in Earth System Models (ESMs), Land Surface Models (LSMs), Gravity Recovery and Climate Experiment (GRACE) data application^1^, and other groundwater related studies^2,3^ (the key abbreviations used in this study are listed in Table 1). Particularly, besides its significance in groundwater modeling, *S*_y_ is also of crucial importance for revealing the global distribution of groundwater resources, as well as enhancing the climate change studies, because groundwater is a vital component of climate system and is essential in sustaining the global adaptation to climate variability and changes^4–6^. However, uncertainties in *S*_y_ determinations are a primary source of error in water table simulations^7–16^.

**Table 1:** Key abbreviations used in this study.

GWSC	Groundwater storage change
GWLC	Groundwater level change
ESMs	Earth System Models
LSMs	Land Surface Models
FAO-UNESCO	FAO and United Nations Educational Scientific and Cultural Organization
GRACE	Gravity Recovery and Climate Experiment data
CC	Correlation coefficient
RSD	Ratio of the standard deviation to the reference
RMSE	Unbiased root mean square error
GSDE	30 seconds Global Soil Dataset for Earth System Models
SoilGrids	SoilGrids 1-km product
HWSD	0.5 degrees Harmonized World Soil Database V1.2
*S*_y_	Specific yield
GASY	Gridded average specific yield produced by this study
GASY–GSDE	Gridded average specific yield based on GSDE
GASY–SoilGrids	Gridded average specific yield based on SoilGrids
GASY–HWSD	Gridded average specific yield based on HWSD
GASY–mean	Mean values of GASY–SoilGrids and GASY–GSDE
GLOBGM–*S*_y_	30 seconds specific yield data, an input to the model of GLOBGM v1.0

*S*_y_ varies spatially and temporally, and is primarily influenced by soil texture, water table depth, and duration^17^, while also affected by other factors such as the temperature and chemical composition of water, the antecedent soil moisture condition, etc. Specifically, according to Johnson^18^, *S*_y_ typically ranges from 0 to 0.5 for different soil textures, with coarser textures yielding higher values. Since the water in the soil medium does not move instantaneously, the shorter the accumulated duration, the smaller the *S*_y_ value, and in this case, it is called as the transient *S*_y_ which is usually estimated through pumping tests or other field methods^17^. Moreover, the soil near the water table will first reach the static equilibrium state or drain completely, because the capillary fringe can supply sufficient water for the soil medium near the water table. It would take a long period of time, potentially several months or years, to reach static equilibrium or drain completely for one soil column, e.g., it would take more than one year for a 1.71-m-long medium-sand column to reach static equilibrium, but the equilibrium state in the bottom 0.6 m can be attained after five hours^19^. When sufficient time is allowed to drain completely or reach the static equilibrium state, *S*_y_ depends on water table depth due to the effect of capillary fringe, known as the apparent *S*_y_. Once the water table reaches a certain depth, related to soil texture, such as 1 m for sand^10,20^, *S*_y_ stabilizes at a ultimate value (a constant for a soil type), and it is referred to as the ultimate *S*_y_. Note that, the ultimate *S*_y_ is obtained only when the water table is deep enough and sufficient time is allowed to reach the static equilibrium condition or drain completely, and the ultimate *S*_y_ is typically determined through laboratory experiments, equating to porosity minus specific retention^18,21–24^. Consequently, due to the high spatiotemporal variability, for the same soil type, the *S*_y_ values under different situations (water table depth, time duration, etc) estimated by different methods vary considerably, even for a given study site. However, the *S*_y_ estimation remains largely uncertain due to methodological limitations, with no widely accepted approach, particularly at a regional scale^25–27^. Moreover, using point-scale *S*_y_ data to represent regional-scale variations is challenging, due to the absence of spatially distributed *S*_y_ data, especially for global simulations. According to the authors’ knowledge, there is no recognized global spatially-distributed *S*_y_ data publicly available to date, and we can only find one set of global *S*_y_ which unfortunately is not published data and just serves as inputs to the GLOBGM model^28^ itself (details in the following section of Methods). Due to these challenges, *S*_y_ determinations in LSMs and ESMs are simplified, introducing uncertainties in groundwater simulations. A uniform empirical constant is commonly used for convenience in global simulations, for example, models like MATSIRO-GW, Noah-MP, and CLM utilize a constant specific yield, but it fails to capture the variability among soil textures worldwide. To address these challenges, building on previous work by Lv *et al*.^17^, this study aims to produce a global dataset of spatially distributed *S*_y_ based on Johnson’s^18^ trilinear graph and three popular soil texture datasets. Because the existing soil texture datasets are all depth-limited, the global *S*_y_ data obtained based on them are correspondingly depth-limited, but readers can expand it into deeper soils by making a reasonable assumption about the vertical variation of soil texture with depth, since the *S*_y_ value is directly and positively correlated with the sand content and negatively correlated with clay content. It shows promise in generating a reference dataset of global gridded *S*_y_, thereby improving future modeling of groundwater dynamics in ESMs/LSMs, and benefiting for understanding the groundwater resources distribution and mitigation of climate change impacts.

## Methods

### Performance metrics

In order to assess the match degree between the global *S*_y_ data produced in this study and the existing references results, this study used three evaluation indices. They are the correlation coefficient (CC), the ratio of the standard deviation to the reference (RSD), the unbiased root mean square error (RMSE).

1 $${\rm{CC}}=\frac{\mathop{\sum }\limits_{i=1}^{N}({x}_{i}-\overline{x})({y}_{i}-\overline{y})}{\sqrt{\mathop{\sum }\limits_{i=1}^{N}{({x}_{i}-\overline{x})}^{2}\mathop{\sum }\limits_{i=1}^{N}{({y}_{i}-\overline{y})}^{2}}}$$

2 $${\rm{RSD}}=\sqrt{\mathop{\sum }\limits_{i=1}^{N}{({x}_{i}-\overline{x})}^{2}/\mathop{\sum }\limits_{i=1}^{N}{({y}_{i}-\overline{y})}^{2}}$$

3 $${\rm{RMSE}}=\sqrt{\frac{\mathop{\sum }\limits_{i=1}^{N}{({x}_{i}-{y}_{i})}^{2}}{N}}$$

where ${x}_{i}$ and ${y}_{i}$ respectively represent the data that need to be validated and the reference data; $\overline{x}$ and $\overline{y}$ denote the averages of ${x}_{i}$ and ${y}_{i}$, respectively; and *N* is number of the data points.

### The trilinear graph

**Fig. 1:**
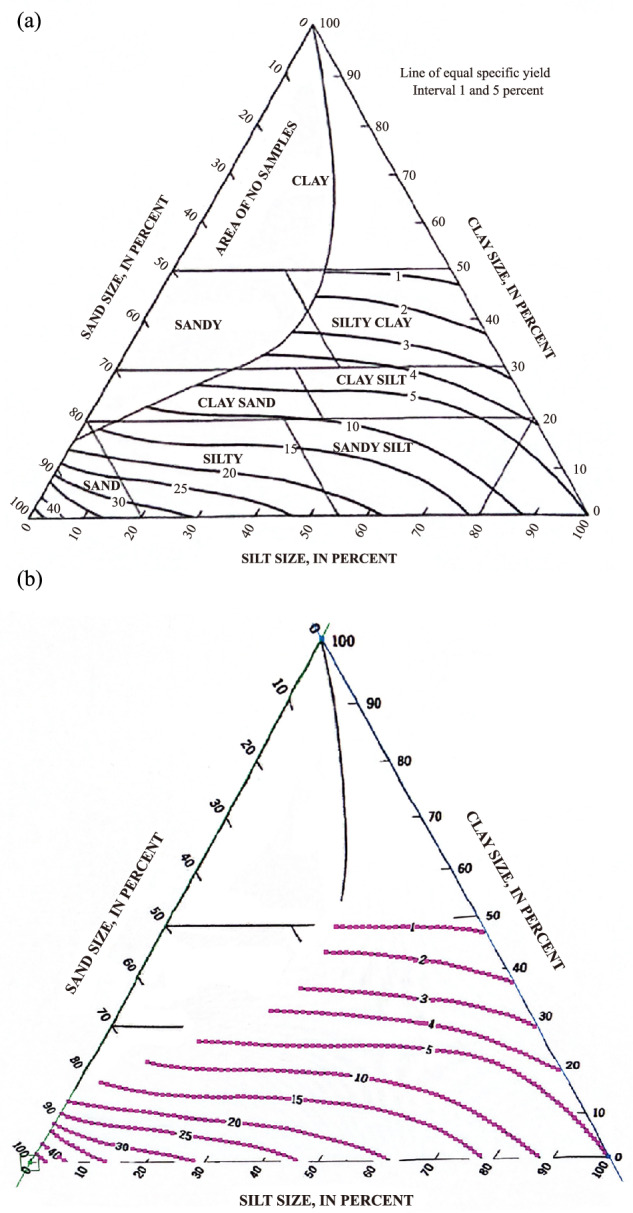
The trilinear graph of Johnson (**a**) and the data points automatically extracted by the GetData Graph Digitizer software (**b**).

The trilinear graph, introduced by Johnson^18^, elucidates the correlation between *S*_y_ values and soil texture classifications based on sand, clay, and silt percentages (Fig. 1a). Comprehensive details regarding this graph can be found in our prior work by Lv *et al*.^17^. Note that, as above-introduced that all methods have limitations, for the trilinear graph, it compiled *S*_y_ values obtained by various methods, but with no attention paid to the effects of soil compaction, anisotropy, local heterogeneity, stratigraphic heterogeneity, the chemical composition of water, etc. Overall speaking, with knowledge of just two of the sand, clay, and silt percentages, one can determine the corresponding *S*_y_ value using this graph, rendering it a cost-effective, time-saving, and user-friendly tool^17^. For instance, Richey *et al*.^29^ utilized it to estimate *S*_y_ for 37 groundwater aquifers globally.

Importantly, the *S*_y_ values used to construct this trilinear graph were derived from both laboratory and field methods^18^ under different situations of water table depth, time duration, heterogeneity, etc, indicating that Johnson’s^18^ trilinear graph likely represents an average value for a given soil texture type, falling between the ultimate *S*_y_ and the transient *S*_y_. Consequently, Johnson’s^18^ trilinear graph holds promise for creating a reference dataset of Gridded Average Specific Yield (hereinafter GASY) for all soil texture types. Interested readers can delve into Johnson’s^18^ study for further insights. With these advantages in mind, we employed this trilinear graph in our study to generate the gridded average *S*_y_ globally.

**Fig. 2:**
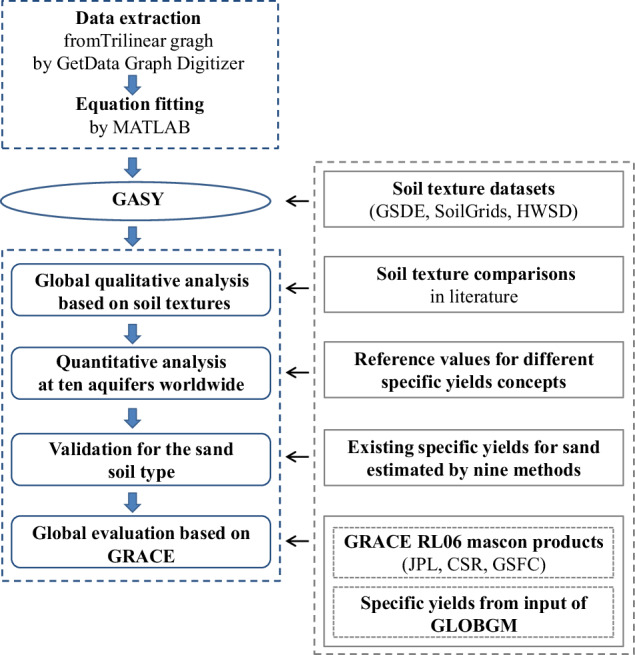
Flowchart of this study.

We first used the GetData Graph Digitizer software to automatically extract the trilinear graph’s *S*_y_ values under the sand and silt/clay axes (Fig. 1b), and then employed multiple linear regression to establish the equation linking the extracted *S*_y_ values with the sand and silt percentages. It was found that, the optimal choice of fitting formula is to first use a fifth-order polynomial (Appendix 1(a)) to perform the global *S*_y_ calculation, and subsequently recalculate the values that fall outside the reasonable range of 0 to 0.5 using a third-order polynomial (Appendix 1(b)). The resulting fitted equations demonstrated a high CC of 0.997 with the extracted data points. Leveraging the fitted equations and global soil texture data, we created the GASY data for different soil textures on a global scale. The conceptual flow chart of this study is provided in Fig. 2. Interested readers can also use the trilinear graph in conjunction with artificial intelligence, e.g., the artificial neural networks^30^.

### Datasets used in validation

To conduct a comprehensive validation and comparison with existing *S*_y_ data in the literature, and to address the requirements of different LSMs or ESMs with multi-layered soil representations, we selected three current widely-used soil texture datasets providing information for multiple layers. These datasets are the 30 seconds Global Soil Dataset for Earth System Models (GSDE)^31,32^, the SoilGrids 1-km product (hereinafter SoilGrids)^33,34^, and the 0.5 degree Harmonized World Soil Database V1.2 (HWSD)^35^, which make it also possible to assess the impact of spatial resolution. The HWSD amalgamates regional and national updates of soil information globally, along with data from the FAO and United Nations Educational Scientific and Cultural Organization (FAO-UNESCO) Digital Soil Map of the World, providing topsoil (0–30 cm) and subsoil (30–100 cm) texture data. The GSDE is derived from an enhanced mapping framework of the HWSD, and it extends the depth and number of soil layers relative to the HWSD, offering eight soil layers with a total depth of 2.296 m. The depths for these eight layers are 0.045, 0.091, 0.166, 0.289, 0.493, 0.829, 1.383, and 2.296 m, respectively. On the other hand, the SoilGrids, developed by the ISRIC using the Global Soil Information Facilities, provides data at seven depths of 0, 0.05, 0.15, 0.3, 0.6, 1, and 2 m. The *S*_y_ data corresponding to these datasets are labeled as GASY–GSDE, GASY–SoilGrids, and GASY–HWSD, respectively. It is important to note that readers have the flexibility to choose their preferred soil texture data based on their specific research requirements.

**Fig. 3:**
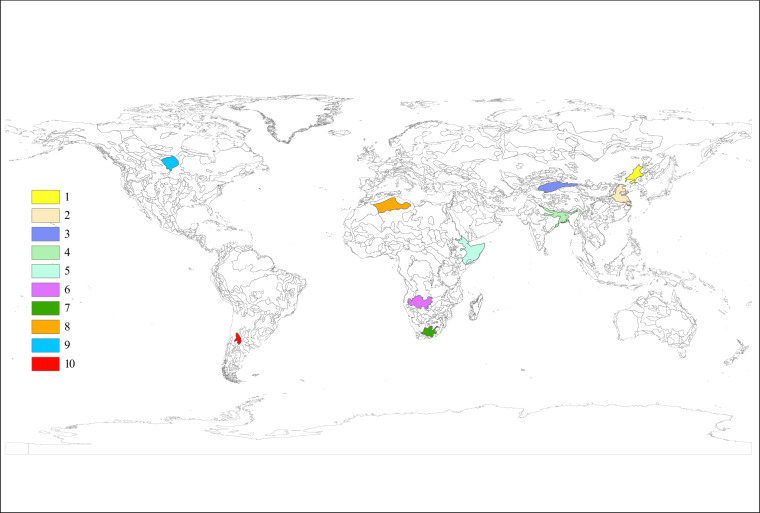
Geographical locations of ten aquifers.

To validate the GASY, we started with a reasonability analysis of spatial distribution for its three sub-datasets on a global scale. Following this, we primarily validated the GASY with reference values derived from various methods for different *S*_y_ concepts across ten groundwater aquifers worldwide (Fig. 3). The selection of study aquifers prioritized those containing minimal soil type variations, thus predominantly representing a single soil texture type. This approach facilitated direct comparison between the GASY and the existing reference results estimated for different *S*_y_ concepts, obtained by laboratory experiments and field observations, with these comparisons tailored to the specific soil texture types, as depicted in Tables 2–4. The predominant soil type for each aquifer was determined by calculating the area-weighted average of sand, silt, and clay percentages. Similarly, the area-averaged GASY value was considered as the *S*_y_ for each respective aquifer.

**Table 2:** Validation of the 0.3–1 m results from GASY–HWSD with current estimations for different concepts obtained by diverse methods in ten aquifers and their statistics indices (“ /“ represents that there is no specific yield value, and “–“ indicates that the index is not calculated).

Aquifer number	Soil type/Evaluation Indices	GASY–HWSD	GLOBGM–*S*_y_	Average *S*_y_ from trilinear graph of Johnson^18,29^	Typical *S*_y_^18,47^	Utimate *S*_y_^47,52–54^	Apparent *S*_y_^47^	Readily available *S*_y_^47^	Aquifer pumping test^48^	Aquifer pumping test^49^
1	Loam	0.099	0.202	0.165	0.095	0.352	0.190	0.075	0.09	/
2	Loam	0.085	0.199	0.165	0.095	0.352	0.190	0.075	0.06	0.075
3	Sandy loam	0.230	0.213	0.225	0.190	0.361	0.290	0.170	/	/
4	Clay loam	0.060	0.203	0.070	0.038	/	0.078	0.021	/	/
5	Sandy clay loam	0.081	0.122	0.085	0.050	/	0.170	0.072	/	/
6	Loamy sand	0.310	0.262	/	0.260	0.353	0.340	0.260	/	/
7	Sandy clay loam	0.057	0.046	0.085	0.050	0.290	0.170	0.072	/	/
8	Clay loam	0.071	0.141	0.070	0.038	0.315	0.078	0.021	/	/
9	Clay loam	0.058	0.088	0.070	0.038	0.315	0.078	0.021	/	/
10	Sandy loam	0.167	0.227	0.225	0.190	0.361	0.290	0.170	/	/
For GASY–HWSD	CC	—	—	0.988	0.965	0.648	0.886	0.958	—	—
	RSD	—	—	0.976	1.076	3.471	0.913	1.090	—	—
	RMSE	—	—	0.011	0.028	0.193	0.078	0.035	—	—
For GLOBGM-*S*_y_	CC	—	—	0.484	0.747	0.940	0.619	0.641	—	—
	RSD	—	—	1.003	0.847	2.759	0.719	0.858	—	—
	RMSE	—	—	0.057	0.083	0.153	0.073	0.096	—	—

**Table 3:** The same as Table 1 but for GASY–GSDE.

Aquifer number	Soil type/Evaluation Indices	GASY–GSDE	GLOBGM–*S*_y_	Average *S*_y_ from trilinear graph of Johnson^18,29^	Typical *S*_y_^18,47^	Utimate *S*_y_^47,52–54^	Apparent *S*_y_^47^	Readily available *S*_y_^47^	Aquifer pumping test^48^	Aquifer pumping test^49^
1	Loam	0.101	0.202	0.165	0.095	0.352	0.190	0.075	0.09	/
2	Loam	0.091	0.199	0.165	0.095	0.352	0.190	0.075	0.06	0.075
3	Sandy loam	0.231	0.213	0.225	0.190	0.361	0.290	0.170	/	/
4	Clay loam	0.051	0.203	0.070	0.038	/	0.078	0.021	/	/
5	Sandy clay loam	0.075	0.122	0.085	0.050	/	0.170	0.072	/	/
6	Loamy sand	0.237	0.262	/	0.260	0.353	0.340	0.260	/	/
7	Sandy clay loam	0.058	0.046	0.085	0.050	0.290	0.170	0.072	/	/
8	Sandy loam	0.199	0.141	0.225	0.190	0.361	0.290	0.170	/	/
9	Clay loam	0.075	0.088	0.070	0.038	0.315	0.078	0.021	/	/
10	Sandy loam	0.139	0.227	0.225	0.190	0.361	0.290	0.170	/	/
For GASY–GSDE	CC	—	—	0.990	0.943	0.685	0.890	0.917	—	—
	RSD	—	—	1.083	0.888	2.734	0.786	0.922	—	—
	RMSE	—	—	0.016	0.027	0.187	0.092	0.033	—	—
For GLOBGM–*S*_y_	CC	—	—	0.374	0.654	0.854	0.532	0.562	—	—
	RSD	—	—	0.857	0.842	2.818	0.747	0.876	—	—
	RMSE	—	—	0.059	0.078	0.159	0.085	0.088	—	—

**Table 4:** The same as Tables 1 and 2 but for GASY-SoilGrids.

Aquifer number	Soil type/Evaluation Indices	GASY–SoilGrids	GLOBGM–*S*_y_	Average *S*_y_ from trilinear graph of Johnson^18,29^	Typical *S*_y_^18,47^	Utimate *S*_y_^47,52–54^	Apparent *S*_y_^47^	Readily available *S*_y_^47^	Aquifer pumping test^48^	Aquifer pumping test^49^
1	Clay loam	0.068	0.202	0.070	0.038	0.315	0.078	0.021	0.09	/
2	Clay loam	0.059	0.199	0.070	0.038	0.315	0.078	0.021	0.06	0.075
3	Loam	0.080	0.213	0.165	0.095	0.352	0.190	0.075	/	/
4	Clay loam	0.038	0.203	0.070	0.038	/	0.078	0.021	/	/
5	Sandy clay loam	0.065	0.122	0.085	0.050	/	0.170	0.072	/	/
6	Sandy Loam	0.162	0.262	0.225	0.190	0.361	0.290	0.170	/	/
7	Sandy clay loam	0.063	0.046	0.085	0.050	0.290	0.170	0.072	/	/
8	Sand	0.288	0.141	0.300	0.340	0.385	0.380	0.320	/	/
9	Clay loam	0.062	0.088	0.070	0.038	0.315	0.078	0.021	/	/
10	Sandy clay loam	0.070	0.227	0.085	0.050	0.290	0.170	0.072	/	/
For GASY–SoilGrids	CC	—	—	0.977	0.990	0.835	0.908	0.977	—	—
	RSD	—	—	0.942	0.760	2.343	0.741	0.800	—	—
	RMSE	—	—	0.025	0.025	0.203	0.085	0.026	—	—
For GLOBGM–*S*_y_	CC	—	—	0.163	0.101	0.293	0.076	0.023	—	—
	RSD	—	—	0.835	0.689	2.148	0.671	0.725	—	—
	RMSE	—	—	0.101	0.133	0.152	0.112	0.137	—	—

Furthermore, the existing global *S*_y_ data (30 seconds), as an input to the model of GLOBGM v1.0 (hereafter referred as GLOBGM–*S*_y_) that is the successor of PCR-GLOBGB 2 (PCRaster Global Water Balance model) groundwater model based on MODFLOW^28^, was also incorporated into the GASY validations in the ten aquifers mentioned above and in simulating global GWLC based on terrestrial water storage changes data. The monthly total terrestrial water storage changes utilized here are the average of GRACE RL06 mascon products of JPL (Jet Propulsion Laboratory)^36^, CSR (Center for Space Research)^37^, as well as the GSFC data^38^ that is comparable to the JPL and CSR mascon products. The monthly land water content changes data from GLDAS-Noah (Global Land Data Assimilation System-Noah 2.7.1)^39^ was also employed in this study, which presents the terrestrial water storage changes but excluding groundwater, and is directly comparable to what GRACE measures over land. Therefore, the GWSC can be obtained by subtracting the GLDAS-Noah water storage change from that of GRACE. According to the data availability, in this study, the GWSC was taken as the multi-year average groundwater storage for July minus the multi-year result for January during the period of 2004 to 2010. Moreover, in the global validation based on GRACE, the datasets of GASY, GLOBGM–*S*_y_, and GRACE were all resampled to the spatial resolution of 1 degree as GLDAS-Noah to ensure consistency in spatial resolution. The corresponding GWLC can then be obtained by dividing GWSC by *S*_y_. Additionally, we assessed the performance of GASY in characterizing *S*_y_ for the sand soil type that has been extensively studied in the literature, through summarizing the existing *S*_y_ values estimated by diverse methods in different areas across the globe.

## Data Records

The GASY data is available at the Zenodo platform^40^ and is publicly accessible via https://zenodo.org/uploads/14216083, with format of Netcdf. The spatial resolution is 30 seconds for the GASY–GSDE, 1 km for the GASY–SoilGrids, and 0.5 degree for the GASY–HWSD. There are two files for the GASY–GSDE, which store the *S*_y_ data for the first four and last four soil layers, respectively. Similarly, there are two files for the GASY–HWSD, corresponding to the top and sub soil layers. For the GASY–SoilGrids, there are seven files, each storing data for one of the seven layers. Each file contains a single variable of *S*_y_.

## Technical Validation

### Reasonability analysis of global spatial distribution

The sub-datasets of GASY (GASY–GSDE, GASY–SoilGrids, and GASY–HWSD) can be derived using the fitted equations obtained from the trilinear graph as described in the Methods section. Because the GASY data were obtained directly according to the soil textures, we observed that all the GASY sub-datasets well reflect the spatial distributions of their corresponding sand content data. For example, comparing the GASY–HWSD with the sand content data from HWSD for the sub-layer (Fig. S1), we found that the areas with higher sand contents tend to have larger *S*_y_, corroborating findings from existing literatures^17^. Specifically, in areas like North Africa, where the sand percentages exceed 80%, the GASY–HWSD *S*_y_ values reach 0.28 and higher. Conversely, in areas such as South China and southeastern North America where the sand contents range from 20% to 50%, their *S*_y_ values are less than or equal to 0.04. Moreover, the GASY–GSDE and the GASY–SoilGrids both present spatial CCs of 1 for all soil layers, while the GASY–HWSD shows the CCs of 0.829 and 0.855 for the sub and top layers, implying that the spatial correlation may be dependent on the spatial resolution.

**Fig. 4:**
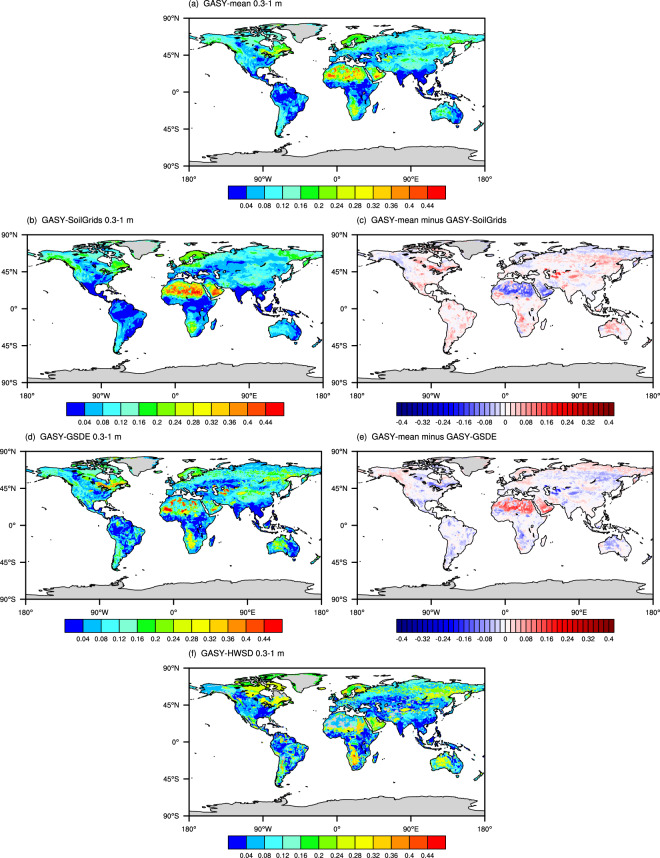
Spatial distributions of GASY–mean (**a**), three sub-datasets of GASY (**b,d,f**), and differences between GASY–mean and GASY–SoilGrids (**c**), GASY–mean and GASY–GSDE (**e**).

To provide a clear view of the GASY sub-datasets, we presented the GASY’s thickness-weighted average over 0.3–1 m depths in Fig. 4, with all data interpolated to the spatial resolution of 0.5 degree of GASY–HWSD. Note that in the GASY–HWSD, missing values exist in many patchy areas, especially in North Africa and West Asia. Previous researches have highlighted substantial local discrepancies in soil texture among commonly used global soil datasets^41,42^. Consequently, it was explainable that the variations among the three sub-datasets of GASY were also apparent at these local areas, since the GASY was directly derived from soil texture data.

Specifically, for instance, a comparison of global clay content distributions from the HWSD, GSDE, and SoilGrids by Stoorvogel & Mulder^43^ revealed localized variations, and from which it was found that the GASY generally displays opposing spatial characteristics to those of clay data, and this result is consistent with existing knowledge that the GASY is negatively correlated with clay content while positively correlated with the sand content. Furthermore, Zhang *et al*.^42^ noted that the sand content in southern Africa, in descending order, is highest in the HWSD, followed by the GSDE, and then the SoilGrids, which is also reflected by the GASY sub-datasets (Fig. 4). The fact that the variations among the GASY sub-datasets are resulted from the soil texture differences among the GSDE, HWSD, and SoilGrids, can be proved again by the spatial patterns of sand content differences. The spatial patterns of differences between any two sub-datasets of the GASY are generally consistent with those of their corresponding sand differences (Fig. S2), with the CCs ranging from 0.719 to 0.799.

The GASY–GSDE aligns more closely with the GASY–HWSD, with the former showing slightly higher *S*_y_ in the mid-latitude region and slightly lower values elsewhere (Fig. 4d,f). This alignment can be attributed to the similarity between the soil textures classified in the HWSD and GSDE, since the GSDE, as an improvement of the HWSD, shares similar soil data sources and mapping approaches with the HWSD^31,42,44^. The evident disparity between the GASY–SoilGrids and the GASY–GSDE/GASY–HWSD presented in North Africa (Fig. 4b,d,f), with the GASY–SoilGrids demonstrating notably higher values compared to the other two sub-datasets, for which a possible explanation may be that the SoilGrids processing smoothed out the extreme values of silt and clay soil samples across all soil layers^45^, and furthermore, it was also found that the sand content from the SoilGrids is larger than the GSDE’s in most of the global land (Fig. S2).

The reasonability analysis of the global spatial distribution suggests that the GASY data are generally reasonable and align with existing knowledge. Readers can choose one of the GASY sub-datasets according to the soil texture data they used, or use the mean values of the GASY–SoilGrids and the GASY–GSDE (hereafter GASY–mean) considering that the GASY–HWSD has a lot of missing values, owns a coarser spatial resolution than the other two datasets, and has similar soil textures with the GASY–GSDE and thus similar *S*_y_ with the GASY–GSDE. Except for the deviation in North Africa, the GASY–mean and the GASY–SoilGrids/GASY–GSDE are only slightly different in the majority of the globe (Fig. 4c,e).

### Quantitative validation at aquifer scale

**Fig. 5:**
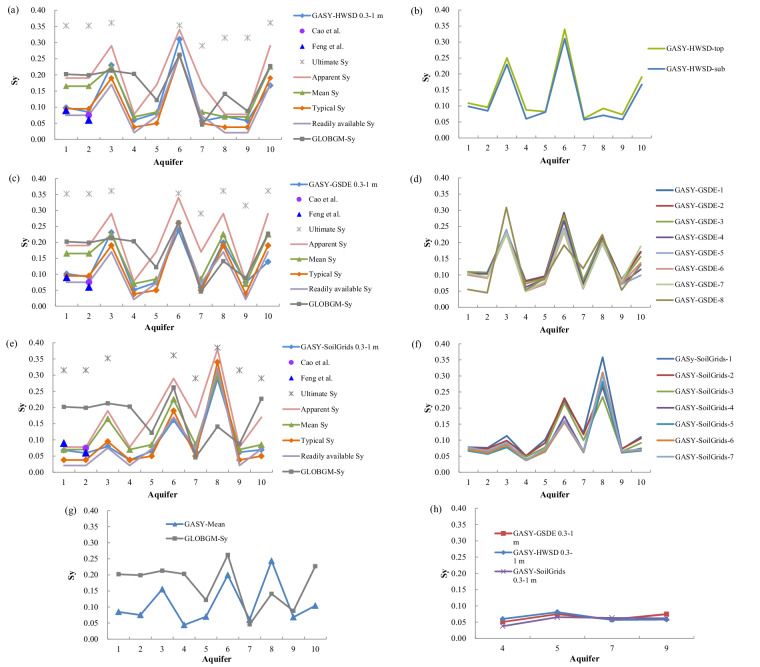
Comparisons of the 0.3–1 m *S*_y_ from three sub-datasets of GASY and existing reference results obtained for different *S*_y_ concepts in ten worldwide aquifers (**a,c,e**); the *S*_y_ values for each soil layer in each sub-dataset of GASY (**d,d,f**); comparison between GASY–mean and GLOBGM–*S*_y_ in these ten aquifers (**g**); and values from three sub-datasets of GASY at aquifers 4, 5, 7, 9 (**h**).

We then conducted a quantitative validation to further evaluate the GASY data in ten aquifers worldwide. Among the reference results, the *S*_y_ from Richey *et al*.^29^ represented average values for different soil textures, directly determined by consulting the trilinear graph of Johnson^18^ based on a soil types map (1 degree) derived from the FAO-UNESCO’s sand, silt, and clay percentages^46^. The typical *S*_y_ reported in literature^18,47^ represented the recognized typical values for different texture classes. Note that the apparent *S*_y_ and the readily available *S*_y_ from Loheide II *et al*.^47^ were both obtained for a water table depth of 1 m. Importantly, the *S*_y_ from the GLOBGM’s global input data were also referenced here to compare with the GASY. Additionally, to ensure a comprehensive validation, the GASY data for aquifers 1 and 2 were also cross-verified with *S*_y_ estimated by the aquifer pumping test method. Figures 5a,c,e illustrates the distinct characteristics of different *S*_y_ concepts as mentioned above: (1) the ultimate *S*_y_, with a constant value, represents the upper limit for the *S*_y_ of a soil texture; (2) the apparent *S*_y_ is usually smaller than the ultimate *S*_y_ but larger than the *S*_y_ estimated using field approaches, such as the aquifer pumping test; and (3) the readily available *S*_y_, estimated using a similar trilinear graph^47^ to that of Johnson^18^ based on diurnal water table fluctuations (about 12 hours), is relatively small compared to the average results.

For the clarity of comparisons, we exclusively present the GASY’s thickness-weighted average over 0.3–1 m depths in these ten aquifers (Fig. 5a,c,e), which is almost comparable to the average for different soil layers (Fig. S3), while the results for different soil layers are illustrated in Fig. 5b,d,f. It is noteworthy that we identified the accuracy of the Richey *et al*.’s^29^ *S*_y_ value for the soil type of loam (at aquifers 1, 2 in Fig. 5a, c, and at aquifer 3 in Fig. 5e) to be questionable, and therefore, the statistical indices here were calculated after excluding this value (Tables 2–4).

Consistent with the finding in the global analysis above that the GASY–GSDE agrees with the GASY–HWSD because of sharing the same soil texture data. The validation in aquifers 1 to 10 reveals that the GASY–GSDE performance closely mirrors the GASY–HWSD, both exhibiting the highest level of consistency with the average *S*_y_ from Richey *et al*.^29^, closely followed by the typical *S*_y_ values reported in literature, characterized by CCs of 0.990–0.943 and 0.988–0.965, RMSEs of 0.016–0.027 and 0.011–0.028, and RSDs of 1.083–0.888 and 0.976–1.076, for the GASY–GSDE and the GASY–HWSD, respectively (Tables 2, and 3). This outcome is explainable as the GASY–GSDE, the GASY–HWSD, and the average *S*_y_ in Richey *et al*.^29^ were all derived based on the trilinear graph^18^ and the FAO-UNESCO soil texture information. Moreover, both the GASY–GSDE and the GASY–HWSD exhibit the third closest to the readily available values from Loheide II *et al*.^47^, while showing worst agreement with the ultimate results. The GASY–GSDE and the GASY–HWSD demonstrate almost identical *S*_y_ values for the aquifers, except in aquifer 8 because the HWSD’s missing data over the area of aquifer 8 resulted in a different soil type with that of GSDE (Tables 2 and 3). Regarding the GASY–SoilGrids, its performance against references demonstrates consistent findings with the GASY–GSDE and the GASY–HWSD, the details can be seen in Fig. 5e and Table 4. The localized differences between the GASY–SoilGrids curve and the other two arise from disparities in their soil textures data, but they align well with each other for the same soil type, e.g., aquifers 4, 5, 7, and 9 where the three GASY datasets share identical soil types (Tables 2–4), which highlights that the GASY production method is robust and sound, and the GASY development is intended for various soil textures but not for specific locations.

The *S*_y_ curves depicted in Fig. 5, alongside Tables 2–4, also demonstrated that the GASY values are more applicable than the GLOBGM–*S*_y_. Specifically, the GLOBGM–*S*_y_ aligns more closely with the ultimate and apparent *S*_y_ relative to the GASY, and as shown in Fig. 5g, the GLOBGM–*S*_y_ curve is apparently higher than that of GASY–mean. Additionally, the GASY *S*_y_ for aquifers 1 and 2 (0.068–0.101 and 0.059–0.091, respectively) are in satisfactory agreement with the aquifer pumping test values from Feng *et al*.^48^ (0.09 and 0.06, respectively) and Cao *et al*.^49^ (0.075 in aquifer 2), while the GLOBGM–*S*_y_ values are too high (0.202 and 0.199, respectively). It is also worth noting that, these pumping test values represent the soil column’s average *S*_y_ weighted by the thickness of the water fluctuation zone^50^, while the *S*_y_ in GASY varies with depth as the soil texture changes vertically and the GASY *S*_y_ values analyzed here (comparable to the average of all soil layers) is the thickness-weighted average over 0.3 to 1 m depths, which accounts for the reasonable agreement of GASY with the pumping test values.

### Validation against existing specific yields for the sand soil type

**Fig. 6:**
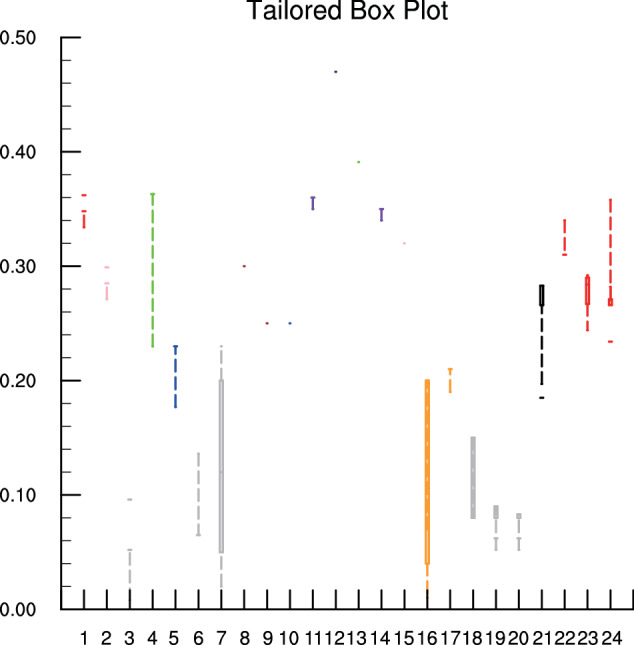
Boxplot of *S*_y_ determined by different methods marked by different colors. Red: The trilinear graph of Johnson^18^, and GASY. Pink: Readily available specific yield. Green: Ultimate specific yield. Blue: Rainfall–water table response method. Gray: Traditional pumping test and slug test. Brown: Laboratory drainage experiment. Purple: Apparent specific yield by the numerical equation of Crosbie *et al*.^7^. Navy: The method provided by Wang & Pozdniakov^55^ based on daily periodic signal. Orange: The water table fluctuation (WTF) method. Black: An improved pumping test method provided by Malama^51^. The abscissa numbers of 1 to 24 present the specific researches, and the details can be found in Table S1.

The GASY dataset was further validated against current *S*_y_ for the soil type of sand, which has been extensively studied in the literature. By compiling *S*_y_ for sand estimated by nine different methods including laboratory experiments and field observations (details provided in Table S1), it was observed that the data from GASY fall within a reasonable range, aligning closely with the average range of results recorded in the literature, as depicted in Fig. 6. This again underscores the GASY’s emergence as a robust global reference dataset for average *S*_y_. Note that the GASY values presented here for sand are taken from those provided in the analysis of the aforementioned ten aquifers.

As described earlier and in the literature, pumping tests typically yield smaller *S*_y_, as indicated by the gray color in Fig. 6. However, it is noteworthy that an improved pumping test method introduced by Malama^51^ yielded larger results compared to older methods, ranging from 0.02 to 0.25 with durations spanning from 15 to 3870 minutes. Importantly, the *S*_y_ values obtained from this improved pumping test method show a better agreement with the GASY results.

### Global validation based on GRACE

**Fig. 7:**
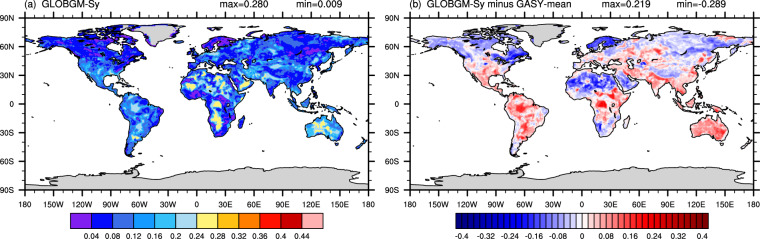
Spatial distribution of GLOBGM–*S*_y_ (**a**) and its difference with GASY–mean (**b**).

At the global scale, the spatial distribution of GLOBGM–*S*_y_ (input to the GLOBGM model) differs significantly from that of GASY-mean (CC of 0.161), especially in the Americas and Asia (Fig. 7). Moreover, neither does it characterize the spatial distribution of soil textures from the GSDE or the SoilGrids, with the CCs of 0.201 and 0.271 with their respective sand content data, respectively. Figure 7 clearly depicted that, relative to the GASY–mean, the GLOBGM–*S*_y_ exhibits positive bias in South America, southern North America, central Africa, Oceania, and most of Asia, which again affirms the conclusions drawn above at the aquifer scale.

**Fig. 8:**
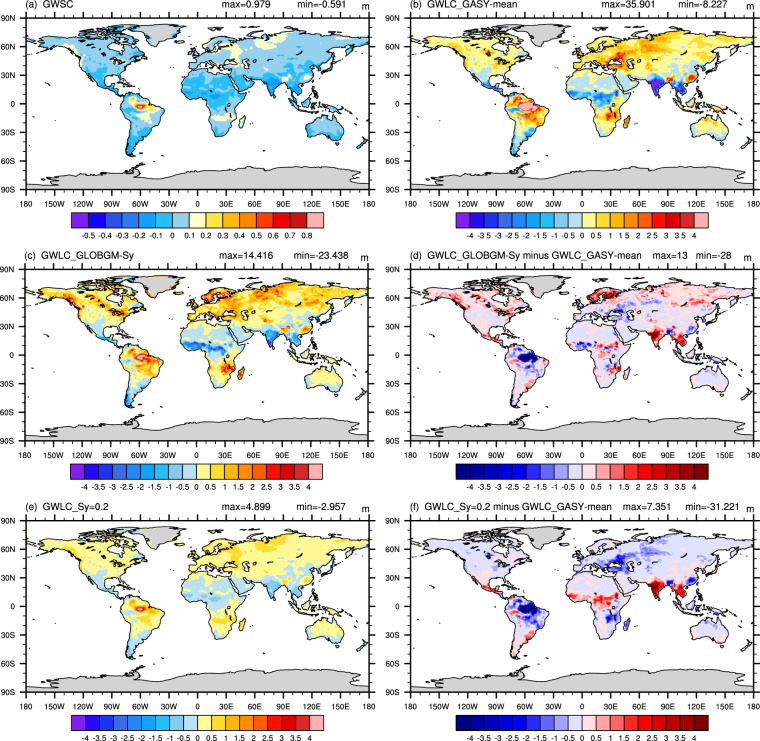
Spatial distributions of GWSC (**a**) and GWLC obtained by GASY–mean, GLOBGM–*S*_y_, and a constant specific yield of 0.2 (**b,c,e**); and spatial maps of difference between GWLC derived by GLOBGM–*S*_y_ or a constant specific yield of 0.2 and that based on GASY–mean (**d,f**).

The global comparison of GWLC derived by dividing GWSC by *S*_y_, revealed that the GWLC obtained by GASY-mean displayed greater spatial correlation with the GWSC (CC of 0.878) than that based on GLOBGM–*S*_y_ (CC of 0.721), while the GWLC calculated using a constant *S*_y_ of 0.2 only captured the spatial characteristic of the GWSC, but failed to reflect the spatial heterogeneity of the soil texture (Fig. 8). The significant GWLC deviations occur mainly at low latitudes near the equator. The maximum positive and negative deviations of the GWLC based on GLOBGM–*S*_y_ from the GWLC obtained by GASY-mean are 13.14 and −28.48, respectively (Fig. 8d), and those for the GWLC based on the constant *S*_y_ are 7.35 and −31.22, respectively (Fig. 8f). Although the GWLC deviations in the rest of world are relatively small as shown in Fig. 8, they will be increasingly large along with the step by step calculation in ESMs and LSMs.

In this study, the validation of the GASY data primarily focused on different soil types, given that the GASY was directly estimated based on soil textures. It is important to note that, for each soil type, it encompasses a range of sand, silt, and clay percentages, resulting in a corresponding range of *S*_y_ values. Consequently, the *S*_y_ for a given soil type may vary across different regions due to differences in the proportions of sand, silt, and clay.

Moreover, it is worth mentioning that the trilinear graph developed by Johnson^18^ has limited samples over the clay and sandy clay areas. As a result, the fitted equations based on this graph may not be directly applicable to clay and sandy clay soil types. Therefore, the applicability of the GASY for clay and sandy clay soils requires further validation in future studies.

Notably, the three soil texture datasets of HWSD, GSDE, and SoilGrids are all depth-limited (about 0–2 m), and therefore, the GASY data obtained based on them are also depth-limited. If there is a soil texture dataset with deeper depth, we can surely obtain the corresponding GASY data by using the fitted equations of this study. For a region where groundwater dynamics occurs at depths > 2 m below ground surface, interested users can reasonably consider (1) taking the *S*_y_ at the last layer of GASY as the result for the soil > 2 m with the assumption that the soil texture below 2 m is the same as the last layer of GASY, or (2) expanding GASY into the depths > 2 m by establishing a reasonable trend for the vertical variation of soil texture with depth, since the *S*_y_ of GASY is directly and positively correlated with the sand content and negatively correlated with clay content, or (3) calculating the *S*_y_ for the depths > 2 m by the fitted equations in this study when the texture of the deep soil is known.

It is crucial to emphasize that the main goal of this study was to develop a global dataset of gridded average *S*_y_ for various soil textures, by employing the multiple linear regression technique to derive the fitted equation, and utilizing soil texture data from the GSDE, the SoilGrids, and the HWSD. However, users have the flexibility to generate their own gridded *S*_y_ using their preferred fitting technologies and selecting alternative soil texture data with different soil depths they needed based on their specific research objectives.

## Supplementary information

Supplementary Information

## Data Availability

We used the software of GetData Graph Digitizer and MATLAB respectively for extracting and fitting data, with the packaged already available tools in them. No custom code was specifically developed or utilized in this study.
