# Supplementary material for: A global dataset of average specific yield for soils
Source: Sci Data. 2025 Mar 12;12:427. doi: 10.1038/s41597-025-04742-1 (PMC11903785; doi:10.1038/s41597-025-04742-1)
Supplement: Supplementary file 1 — Supplementary Information [file 41597_2025_4742_MOESM1_ESM.pdf]

Supplementary information for

A global dataset of average specific yield for soils

Meizhao Lv[1], Meixia Lv[1], Yuanyuan Zha[4], Lei Wang[2,5*], Zong-Liang Yang[3*]

1. CAS Key Laboratory of Regional Climate and Environment for Temperate East Asia, Institute of Atmospheric Physics, Chinese Academy of Sciences, Beijing, China.
2. State Key Laboratory of Tibetan Plateau Earth System, Environment and Resources, Institute of Tibetan Plateau Research, Chinese Academy of Sciences, Beijing, China.
3. Department of Earth and Planetary Sciences, Jackson School of Geosciences, The University of Texas at Austin, TX 78705, USA.
4. State Key Laboratory of Water Resources Engineering and Management, Wuhan University, Wuhan, China.
5. The University of Chinese Academy of Sciences, Beijing, China.

Contents for this file:

Fig. S1 Spatial distribution of GASY–HWSD (a) and the spatial distribution of HWSD sand content (b) for sub layer.

Fig. S2 Comparisons of the difference between two of GASY sub-datasets and the difference between their corresponding sand contents.

Fig. S3 Curves of the 0.3–1 m specific yield and the layer-averaged specific yield at ten aquifers from GASY–HWSD, GASY–GSDE, and GASY–SoilGrids.

Appendix 1: (a) Fifth-order polynomial fitting equation; (b) Third-order polynomial fitting equation.

Table S1 Validation of GASY with existing specific yields for different concepts determined by nine methods for the soil type of sand.

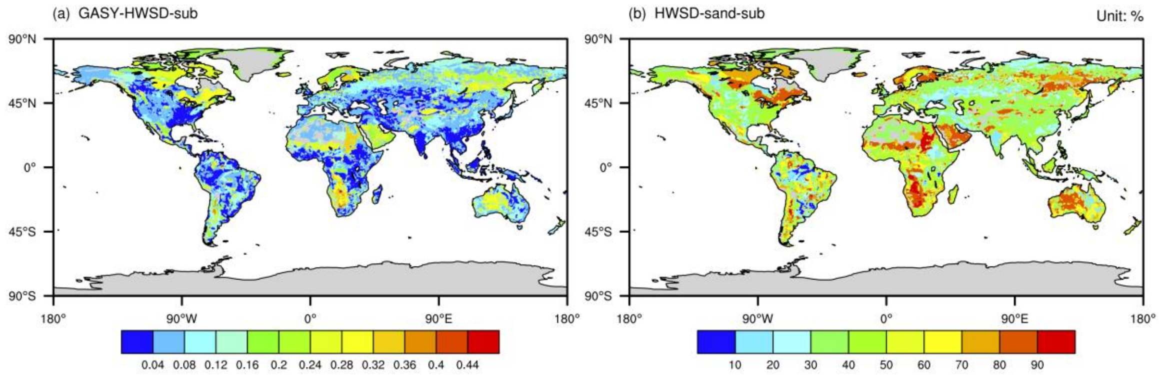

Fig. S1 Spatial distribution of GASY–HWSD (a) and the spatial distribution of HWSD sand content (b) for sub layer.

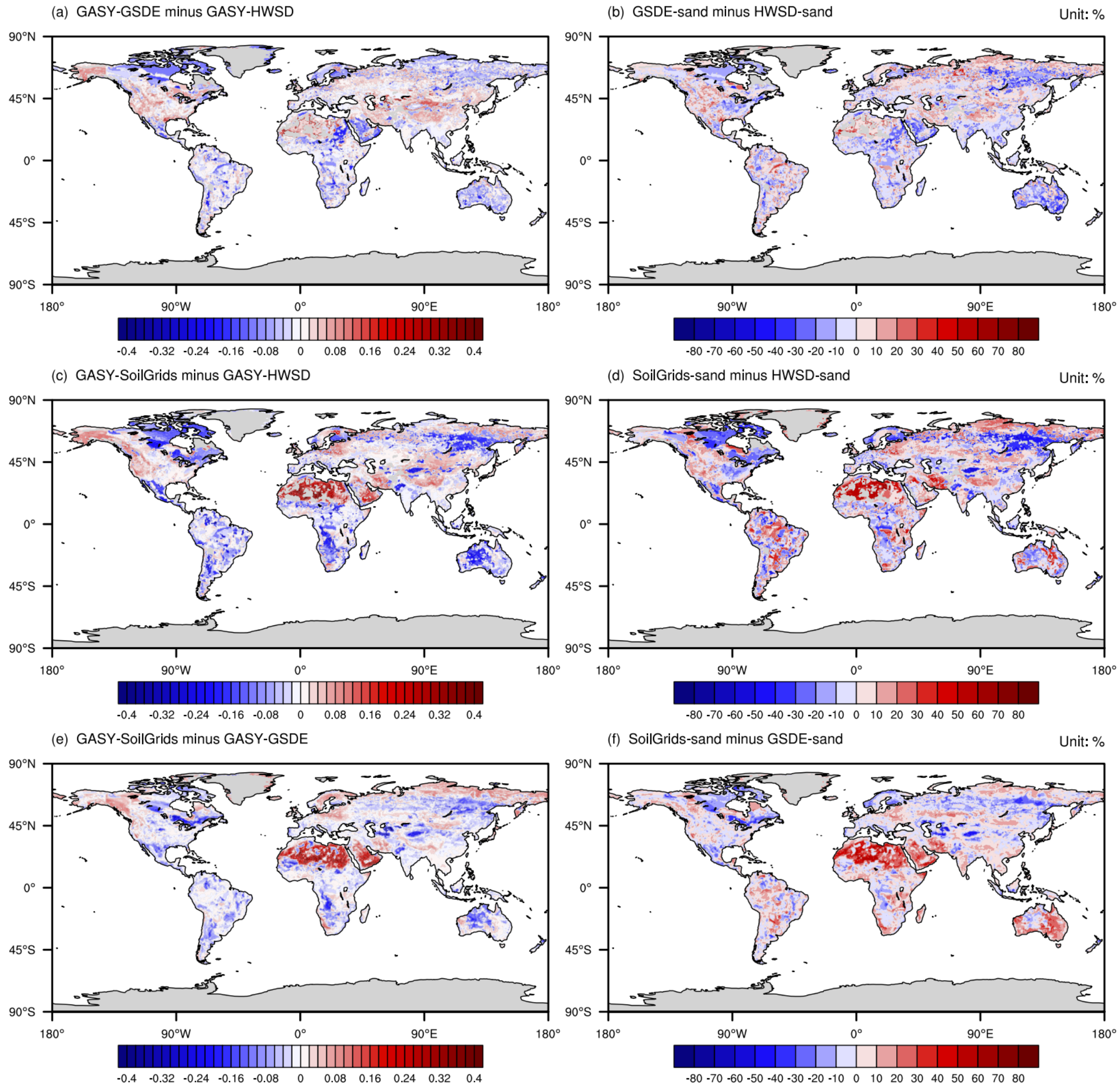

Fig. S2 Comparisons of the difference between two of GASY sub-datasets (a, c, e) and the difference between their corresponding sand contents (b, d, f).

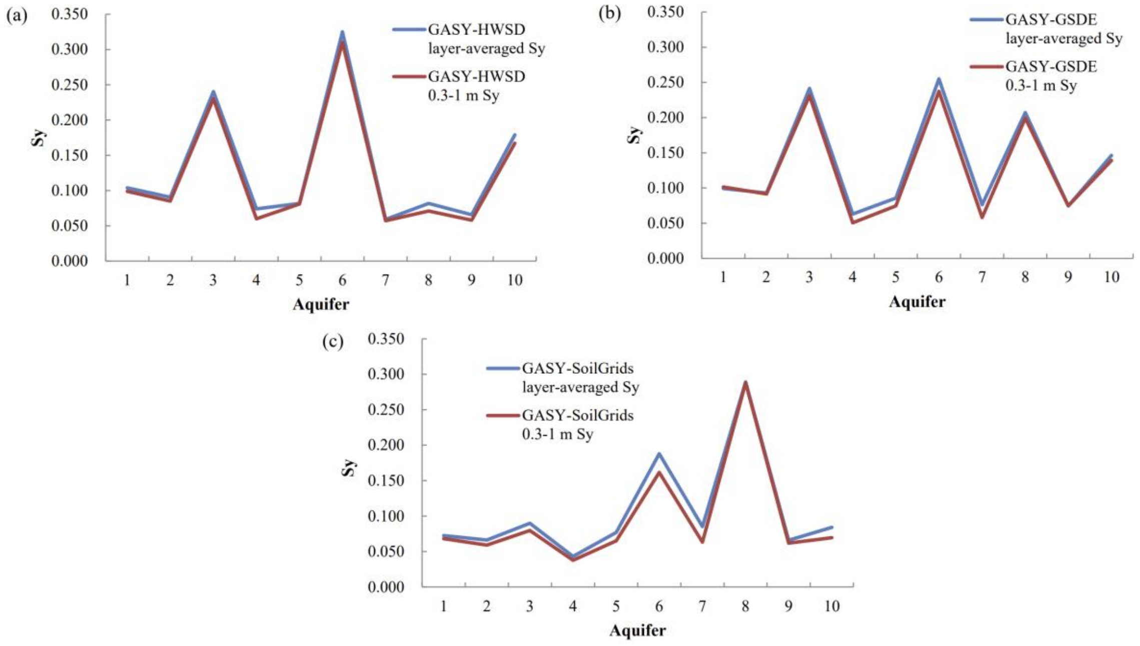

Fig. S3 Curves of the 0.3–1 m specific yield and the layer-averaged specific yield at ten aquifers from GASY–HWSD (a), GASY–GSDE (b), and GASY–SoilGrids (c).

Appendix 1

(a) Fifth-order polynomial fitting equation:

$$S_y = x0+x1+x2+x3+x4+x5+x6+x7+x8+x9+x10+x11+x12+x13+x14+x15+x16+x17$$

$x0 = -1.304+0.06447\times silt+0.01754\times sand$

$x1 = -0.00117\times silt^2$

$x2 = 0.001375\times silt\times sand$

$x3 = 0.001643\times sand^2$

$x4 = 9.388e\text{-}06\times silt^3$

$x5 = -6.835e\text{-}05\times silt^2\times sand$

$x6 = -0.0001356\times silt\times sand^2$

$x7 = -5.048e\text{-}05\times sand^3$

$x8 = -2.782e\text{-}08\times silt^4$

$x9 = 9.14e\text{-}07\times silt^3\times sand$

$x10 = 2.6e\text{-}06\times silt^2\times sand^2$

$x11 = 2.167e\text{-}06\times silt\times sand^3$

$x12 = 5.126e\text{-}07\times sand^4$

$x13 = -3.796e\text{-}09\times silt^4\times sand$

$x14 = -1.418e\text{-}08\times silt^3\times sand^2$

$x15 = -1.854e\text{-}08 \times silt^2 \times sand^3$

$x16 = -1.005e\text{-}08 \times silt \times sand^4$

$x17 = -1.717e\text{-}09 \times sand^5$

where S_y is the specific yield, silt and sand present the silt and sand percentage, respectively, the variables of x0 to x17 are all intermediate variables, with the same meanings for the third-order polynomial fitting equation below.

(b) Third-order polynomial fitting equation:

$S_y = x0+x1+x2+x3+x4+x5+x6+x7$

$x0 = 0.4133-0.01723 \times silt-0.01543 \times sand$

$x1 = 0.0002345 \times silt^2$

$x2 = 0.0003879 \times silt \times sand$

$x3 = 0.0001105 \times sand^2$

$x4 = -9.982e\text{-}07 \times silt^3$

$x5 = -1.664e\text{-}06 \times silt^2 \times sand$

$x6 = -7.329e\text{-}07 \times silt \times sand^2$

$x7 = 4.784e\text{-}07 \times sand^3$

Table S1 Validation of GASY with existing specific yield (S_y) values for different concepts determined by nine methods for the soil type of sand（"$\theta_s - S_r$" represents porosity minus specific retention）

Texture	Study area	Reference	Abscissa number in Fig. 6	Method	S_y	Supplementary information
Sand	A valley location in the Hidegviz Valley experimental catchment in Hungary, with sand and loamy sand.	Gribovszki[1]	1	Trilinear graph of Johnson[2]	0.348 ±0.014	GASY is based on this method
			2	Trilinear graph of Loheide II et al.[3]	0.285 ±0.014	Readily available S_y
			3	Slug test	0.052 ±0.044	Based on van Beers[4]
	Two field sites within the Tomago Sandbeds near Newcastle, Australia, with fine sand.	Crosbie et al.[5]	4	$\theta_s - S_r$	0.230, 0.363	Ultimate S_y at two sites
			5	Rainfall–water table response method	0.177, 0.230	Values at two sites
			6	Type-curve method of Moench[6]	0.136, 0.065	Values at two sites
	A field site in an shallow unconfined medium-grained sand aquifer at the Canadian Forces Base Borden, Ontario. Water table depth of 2.3 m.	Nwankwor et al.[7]	7	Volume-balance method	0.02–0.25	Time duration : from 15 to 3,870 minutes
			8	Laboratory drainage experiment	0.3	Ultimate S_y
	An unconfined surficial aquifer on Bribie Island in Australia, with sandy soil.	Fan et al.[8]	9	Laboratory drainage experiment	0.25	Apparent S_y for more than 1 m water table depth determined by laboratory-based drainage experiments on extracted cores
			10	Rainfall–water table response method	0.25	For the water table depth of more than 1 m
	A study area in Ejina Oasis, northwestern China, with sand. Water table depth of 1–4 m.	Wang & Pozdniakov[9]	11	The numerical equation of Crosbie et al.[5]	0.35–0.36	Apparent S_y
			12	The method provided by Wang & Pozdniakov[9]	0.47	Based on daily periodic signal
			13	$\theta_s - S_r$	0.391	Ultimate S_y.

Two field sites within a sandy aquifer in Ejina Oasis, located in the lower reaches of the Heihe River Basin, China. Water table depth of 2–4m	Wang et al.[10]	14	The numerical equation of Crosbie et al.[5]	0.34，0.35	Apparent S_y for two sites
		15	Trilinear graph of Loheide II et al.[3]	0.32	Readily available S_y when the water table depth is ≥1 m
The Upper Danube catchment of southern Germany and northern Italy, with unconsolidated alluvial sand and gravel deposits. 25 wells with water table depths of around 0.82–81.07 m.	Zhang et al.[11]	16	Water table fluctuation (WTF) method based on water budget equation	0.01–0.25	Values at 25 wells
The Larned Research Site riparian zone with the sand and gravel aquifer, the United States. Water table depth of about 2.13–3.36 m.	Butler Jr. et al.[12]	17	WTF method based on neutron meter and pressure transducers	0.19–0.21	Cited from McKay et al.[13]
The Ordos Plateau in China, with sand and sandstone.	Yin et al.[14]	18	Type-curve method based on Boulton[15], Neuman & Witherspoon[16], and Moench[17]	0.08–0.18	Values at 46 wells
The Biose Hydrogeophysical Research Site in Boise, Idaho (US), with unconsolidated cobble and sand fluvial deposits. Mean water table depth of 2 m.	Malama[18]	19	Type-curve method of Neuman[19]	0.052–0.090	Values at 4 wells. Time duration: 10^3 minutes.
		20	Type-curve method of Moench[20]	0.052–0.083	
		21	The improved type-curve method of Malama[18]	0.185–0.283	
Two aquifers with the soil of sand and loamy sand	GASY	22	GASY–HWSD	0.310–0.340	Values from 2 layers of GASY–HWSD, for aquifer 6 with loamy sand
		23	GASY–GSDE	0.192–0.290	Values from 8 layers of GASY–GSDE, for aquifer 6 with loamy sand
		24	GASY–SoilGrids	0.234–0.311	Values from 7 depths of GASY–SoilGrids, for aquifer 8 with sand

References in Table S1

1. Gribovszki, Z. Comparison of specific-yield estimates for calculating evapotranspiration from diurnal groundwater-level fluctuations. *Hydrogeology Journal* **26,** 869–880 (2018).
2. Johnson, A. I. Specific yield: compilation of specific yields for various materials (No. 1662). (US Government Printing Office, 1967).
3. Loheide II, S. P., Butler Jr, J. J., & Gorelick, S. M. Estimation of groundwater consumption by phreatophytes using diurnal water table fluctuations: A saturated-unsaturated flow assessment. *Water Resources Research* **41,** W07030 (2005).
4. ILRI. Fieldbook for land and water management experts (International Institute for Land Reclamation and Improvement, 1972)
5. Crosbie, R. S., Binning, P., & Kalma, J. D. A time series approach to inferring groundwater recharge using the water table fluctuation method. *Water Resources Research* **41,** W01008 (2005).
6. Moench, A. F. Specific yield as determined by type-curve analysis of aquifer-test data. *Groundwater* **32,** 949–957 (1994).
7. Nwankwor, G., Cherry, J., & Gillham, R. A comparative study of specific yield determinations for a shallow sand aquifer. *Groundwater* **22,** 764–772 (1984).
8. Fan, J., Oestergaard, K. T., Guyot, A., & Lockington, D. A. Estimating groundwater recharge and evapotranspiration from water table fluctuations under three vegetation covers in a coastal sandy aquifer of subtropical Australia. *Journal of Hydrology* **519,** 1120–1129 (2014).
9. Wang, P., & Pozdniakov, S. P. A statistical approach to estimating evapotranspiration from diurnal groundwater level fluctuations. *Water Resources Research* **50,** 2276–2292 (2014).
10. Wang, P. et al. Application of the water table fluctuation method for estimating evapotranspiration at two phreatophyte-dominated sites under hyper-arid environments. *Journal of Hydrology* **519,** 2289–2300 (2014).
11. Zhang, J., van Heyden, J., Bendel, D., & Barthel, R. Combination of soil-water balance models and water-table fluctuation methods for evaluation and improvement of groundwater recharge calculations. *Hydrogeology Journal* **19,** 1487–1502 (2011).
12. Butler Jr., J. J. et al. A field investigation of phreatophyte-induced fluctuations in the water table. *Water Resources Research* **43,** W02404 (2007).
13. McKay, S. E., Kluitenberg, G. J., Butler Jr, J. J., Zhan, X., Aufman, M. S., & Brauchler, R. In-situ determination of specific yield using soil moisture and water level changes in the riparian zone of the Arkansas River, Kansas. *Eos Trans. AGU* **85** (2004).
14. Yin, L. et al. Groundwater-recharge estimation in the Ordos Plateau, China: comparison of methods. *Hydrogeology Journal **19,*** 1563–1575 (2011).
15. Boulton, N. S. Analysis of data from non-equilibrium pumping tests allowing for delayed yield from storage. *Proceedings of the Institution of Civil Engineers* **26,** 469–482 (1963).
16. Neuman, S, P., & Witherspoon, P. A. Field determination of hydraulic properties of leaky multiple aquifer systems. *Water Resources Research* **8,** 1284–1298 (1972).
17. Moench, A. F. Combining the Neuman and Boulton models for flow to a well in an unconfined aquifer. *Groundwater* **33,** 378–384 (1995).
18. Malama, B. Alternative linearization of water table kinematic condition for unconfined aquifer pumping test modeling and its implications for specific yield estimates. *Journal of Hydrology,* **399,** 141–147 (2011).
19. Neuman, S. P. Theory of flow in unconfined aquifers considering delayed response of the water table. *Water Resources Research* **8,** 1031–1045 (1972).
20. Moench, A. F. Flow to a well of finite diameter in a homogeneous, anisotropic water table aquifer. *Water Resources Research* **33,** 1397–1407 (1997).
